# An unmanned aerial vehicle captured dataset for railroad segmentation and obstacle detection

**DOI:** 10.1038/s41597-024-03952-3

**Published:** 2024-12-02

**Authors:** Rampriya R. S., Taher Al-Shehari, Sabari Nathan, Jenefa A., Suganya R., Shunmuga Perumal P., Taha Alfakih, Hussain Alsalman

**Affiliations:** 1 School of Computer Science Engineering and Information Systems, Vellore Institute of Technology, Vellore, 632014 Tamilnadu India; 2 Department of Self-Development Skill, Common First Year Deanship, King Saud University, 11362 Riyadh, Saudi Arabia; 3 Couger Inc, Tokyo, 150-0001 Japan; 4 School of CST, Karunya Institute of Technology and Sciences, Coimbatore, 641114 Tamilnadu India; 5 School of Computer Science and Engineering, Vellore Institute of Technology, Chennai, 600127 Tamilnadu India; 6 Department of Information Systems, College of Computer and Information Sciences, King Saud University, Riyadh, 11543 Saudi Arabia; 7 Department of Computer Science, College of Computer and Information Sciences, King Saud University, Riyadh, 11543 Saudi Arabia

**Keywords:** Engineering, Aerospace engineering, Research data

## Abstract

Safety is crucial in the railway industry because railways transport millions of passengers and employees daily, making it paramount to prevent injuries and fatalities. In order to guarantee passenger safety, computer vision, unmanned aerial vehicles (UAV), and artificial intelligence will be essential tools in the near future for routinely evaluating the railway environment. An unmanned aerial vehicle captured dataset for railroad segmentation and obstacle detection (UAV-RSOD) comprises high-resolution images captured by UAVs over various obstacles within railroad scenes, enabling automatic railroad extraction and obstacle detection. The dataset includes 315 raw images, along with 630 labeled and 630 masked images for railroad semantic segmentation. The dataset consists of 315 original images captured by the UAV for object detection and obstacle detection. To increase dataset diversity for training purposes, we applied data augmentation techniques, which expanded the dataset to 2002 augmented and annotated images for obstacle detection cover six different classes of obstacles on railroad lines. Additionally, we provide the original 315 images along with a script for augmentation, allowing users to generate their own augmented data as needed, offering a more sustainable and customizable option. Each image in the dataset is accurately annotated with bounding boxes and labeled under six categories, including person, boulder, barrel, branch, jerry can, and iron rod. This comprehensive classification and detailed annotation make the dataset an essential tool for researchers and developers working on computer vision applications in the railroad domain.

## Background & Summary

Rail transit remains a crucial transportation method for both people and cargo, serving routes that span short to long distances. Over the last decade, the European Economic Community has experienced a reduction in railway accidents, the fundamental causes of these accidents have remained the same, with a 35.1% decrease in casualties from 2010 to 2022^1^. Among the world’s largest railway networks is Indian Railways, spanning 67,956 kilometers. The Annual Report for 2021 released by Indian Railways documented a total of 35 train incidents during the fiscal year 2021–2022, most of which involved train derailments. Obstacles on the railroad track can lead to derailments, resulting in significant damage to the railroad infrastructure and causing fatalities and injuries. The number of accidents is increasing daily due to various factors, such as fallen trees on the gauge or boulders on the track^2^. Fayyaz *et al*.^3^ have observed that the railroad’s operations can be unpredictable, which has led to an increased demand for a fixed obstacle detection system. They also highlight that static impediments are among the most hazardous elements of the railroad.

With personnel working on railroads, it has been feasible to observe these occurrences. However, in real-world situations like these, the work becomes deadly and necessitates additional workers, especially in hazardous areas such as dense forests, tall bridges, and rural areas^4^. Moreover, manual monitoring is insufficient to prevent derailment incidents. Indian Railways states that negligent railroad workers are to blame for 57% of major train accidents^5^. Therefore, the adoption of advanced technologies is crucial for enhancing passenger safety. It is claimed that by using advanced technologies and frequent supervision, transportation systems can significantly decrease accident rates and improve overall safety.

UAVs were initially used for detecting plants^6^, surveying forests^7^, monitoring traffic^8^, and providing security^9^. UAVs have demonstrated significant effectiveness across various sectors, with their impact being particularly notable in the transportation industry. An intelligent transport system can greatly benefit from the efficient data collection procedure provided by a UAV equipped with a camera^10^. For regular and dynamic railroad condition monitoring, unmanned aerial vehicles (UAVs) equipped with deep learning and aerial image processing capabilities are emerging as a key area of research. Indian Railways has considered using unmanned aerial vehicles (UAVs) to monitor the railway environment and ensure passenger safety due to their low cost and high mobility^11^. Compared to traditional methods, UAVs offer advantages in terms of cost-effectiveness, early detection, accessibility, and increased effectiveness in routine observation. Thus, it is highly recommended to employ intelligent monitoring systems centered on UAVs to continuously and proactively inspect the railway lines.

**Fig. 1:**
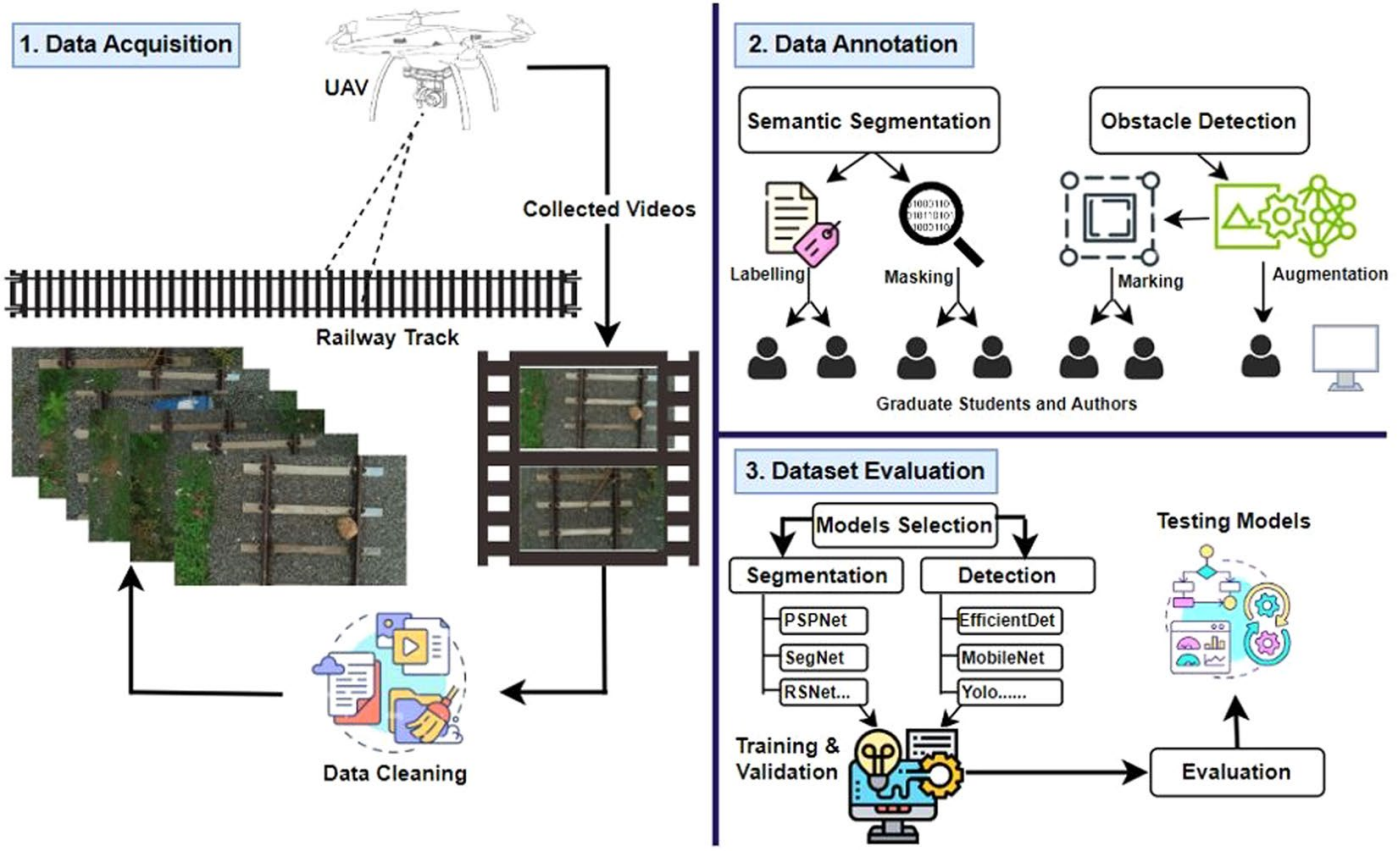
An overview of the work’s pipeline. (**a**) Dataset Acquisition. (**b**) Dataset annotation. (**c**) Dataset Evaluation.

From this vantage point, railroad segmentation and railroad obstacle recognition from aerial photos have become increasingly popular artificial intelligence research topics. Compared to other transportation accidents, railroad accidents are more deadly in terms of severity and fatality rate. Sen *et al*.^12^ state that to maintain passenger safety and execute routine interval track monitoring, sophisticated development and research are necessary. Therefore, recording the railroad with obstacles becomes crucial as a first step in order to gain a thorough view of the railroad catastrophe. As a result, this dataset facilitates the implementation of obstacle identification and semantic segmentation in a real-time railroad environment, guaranteeing precise railroad extraction and obstacle detection. The primary workflow of the UAV-RSOD dataset is depicted in Fig. 1, which includes dataset acquisition, dataset annotation by graduate students and authors, and dataset evaluation. The accuracy of railroad scene segmentation is being improved by a number of groundbreaking datasets, including Cityscapes^13^, Mapillary Vistas^14^, COCO-Stuff^15^, Kitti^16^, and RailSem19^17^. Although semantic segmentation has advanced, the lack of benchmark datasets makes semantic segmentation and object detection following derailments challenging. Moreover, in order to detect obstacles or segment the railroad from the viewpoint of unmanned aerial vehicles, the authors realized that there is no publicly accessible UAV railroad dataset.

Through the use of the visual components included in images, a variety of computer vision algorithms can make a substantial contribution to accurate railroad assessment. Within computer vision, there are various components, including image classification, image segmentation, semantic segmentation, and object detection. Image classification is a process in which a single label or category is assigned to an entire image based on its overall content. In contrast, image segmentation, a more detailed technique within the field of computer vision, involves breaking down an image into multiple segments or groups of pixels. Conversely, stands out as a crucial approach for detailed image understanding. It involves delineating each object within an image down to the pixel level, providing clear and distinct borders. Another computer vision method, object detection, locates and identifies objects in an image or video by drawing bounding boxes around these objects, making it easier to determine their location within a scene. Significant advancements in segmentation and object detection in railroad contexts have recently been made, mostly due to the use of deep learning techniques.

In this work, we develop a diversified dataset called ‘UAV-RSOD’, which is specifically intended for railway semantic segmentation and obstacle identification within the railway setting. UAV-RSOD is an essential resource for developing and testing railroad semantic segmentation and obstacle detection models because it includes a total of 2002 high-resolution images. Based on our current understanding, UAV-RSOD is the first low-altitude UAV-based railroad dataset for semantic segmentation and obstacle detection that is made available to the public. In summary, the following are this paper’s primary contributions:

1. Dataset Establishment: First, videos of railroad lines with various obstacles like boulders, iron rods, persons, jerry cans, barrels, and tree branches are collected using UAVs. Second, data cleaning is performed, which ignores frame duplications and removes noisy or blurred images. Third, data annotation is done through labeling, masking, and bounding box marking for semantic segmentation and obstacle detection, respectively.
2. Dataset Validation: The dataset’s quality and usability are confirmed by technical validation, which involves benchmarking different popular deep learning models. This provides researchers with adequate experimental data for future research and reference.
3. Dataset Accessibility: Researchers and developers can now access the UAV-RSOD dataset as it has been made publicly available. In the areas of railroad semantic segmentation and obstacle detection in railroad environments, this will support research and innovation.

## Methods

### Data collection

**Fig. 2:**
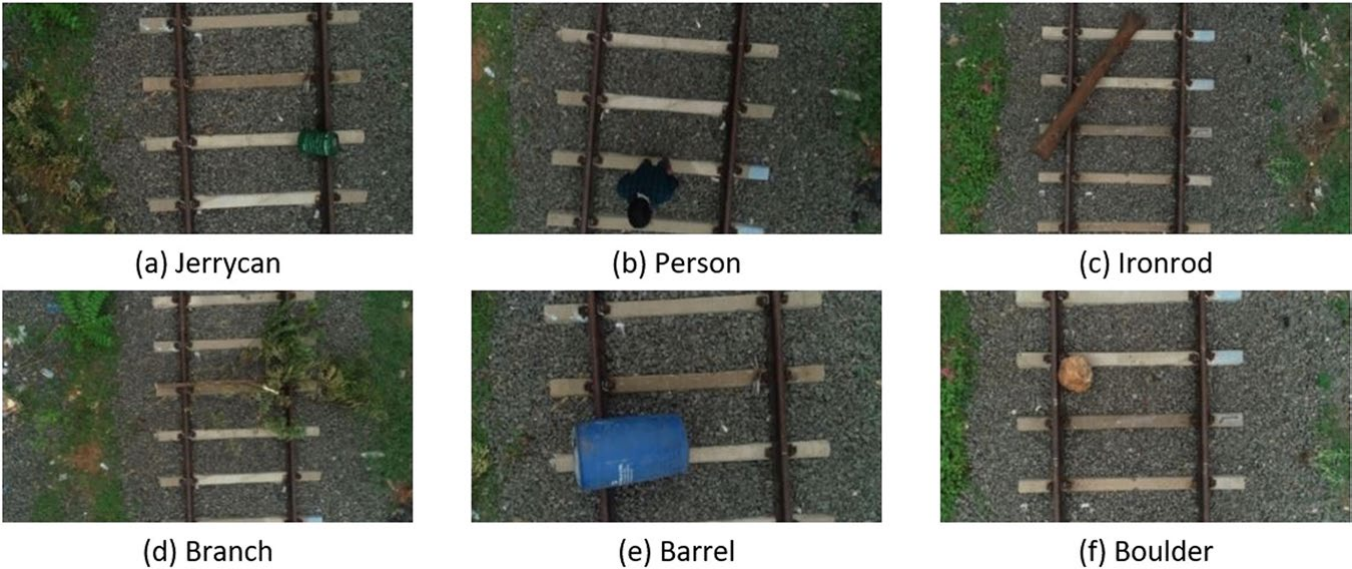
Sample aerial images of railroads in the UAV-RSOD dataset can verify various deep neural network models, with classes (**a**–**f**) representing the dataset.

The research site is the Junction Railway Station located in Tiruchirapalli, Tamil Nadu, India. The train station is situated at an elevation of 95 meters and features 13 tracks and 8 platforms. Among these tracks, one that was not in use was utilized to gather data. In order to set up the rail track field for airborne railroad obstacle identification, various obstacles were considered, including a jerry can, person, iron rod, barrel, branch, and boulder (Fig. 2).

A high-resolution Sony DSC-RX1RM2 camera with 20 megapixels is included with the DJI Phantom 4 PRO UAV for data collection purposes. Table 1 describes the configuration of the Phantom 4 PRO UAV that was employed. The aerial footage is captured using a camera set to an 8.8 mm focal length and 1920 × 1080 HD video recording at 50 Mbps, with the flight route being remotely navigated from the terrain. The research area’s railroad layout is 500 meters long in total. The flying height was set at 10.5 meters above the rail, train, and overhanging lines on the railroad. The Research Design and Standards Organization (RDSO)^18^ in India has established guidelines stating that the average height of overhead electrical lines from the level of the train should be 5.55 meters. This specification was crucial in determining the precise altitude required for accurate data collection in our study.

**Table 1:** DJI Phantom 4 PRO UAV Configuration Details.

Properties	Unit
UAV Brand	DJI Phantom 4 Pro UAV
Weight	1388 g
Max Flight Time	~30 Mins
Obstacle Sensing	Front, Rear, Left & Right Obstacle Avoidance
Vision System	Forward, Backward and Downward
Camera	Sony DSC-RXC1RM2, 20 M full resolution
Resolution	1920 × 1080
Length of railroad	378 m
Flying Altitude	10 m
No. of images	315

On September 15, 2020, we conducted an aerial survey and recorded video footage in .mov format. The footage was carefully captured to ensure it met the necessary standards and provided clear, usable data for our research. Following the acquisition of the aerial footage, it was subsequently uploaded to the annotation mechanism of IBM Cloud^19^. The videos were then downloaded and converted into frames in the.jpg image format, resulting in the creation of 315 images featuring obstacles. Through a variety of geometric transformation techniques, the 315-image dataset was expanded to 2002 images, six times its original size, which are divided into five classes as mentioned previously. The data gathering was carried out in accordance with the 2017 regulations for drone operations in India established by the Directorate General of Civil Aviation (DGCA)^20^. A licensed remote pilot, identified by a unique number issued by the DGCA, was tasked with data collection using UAVs. This is a primary requirement for deploying UAVs in a railway context. Additionally, authorization from Indian Railways division officials was secured to facilitate the necessary data gathering.

### Data annotation

**Fig. 3:**
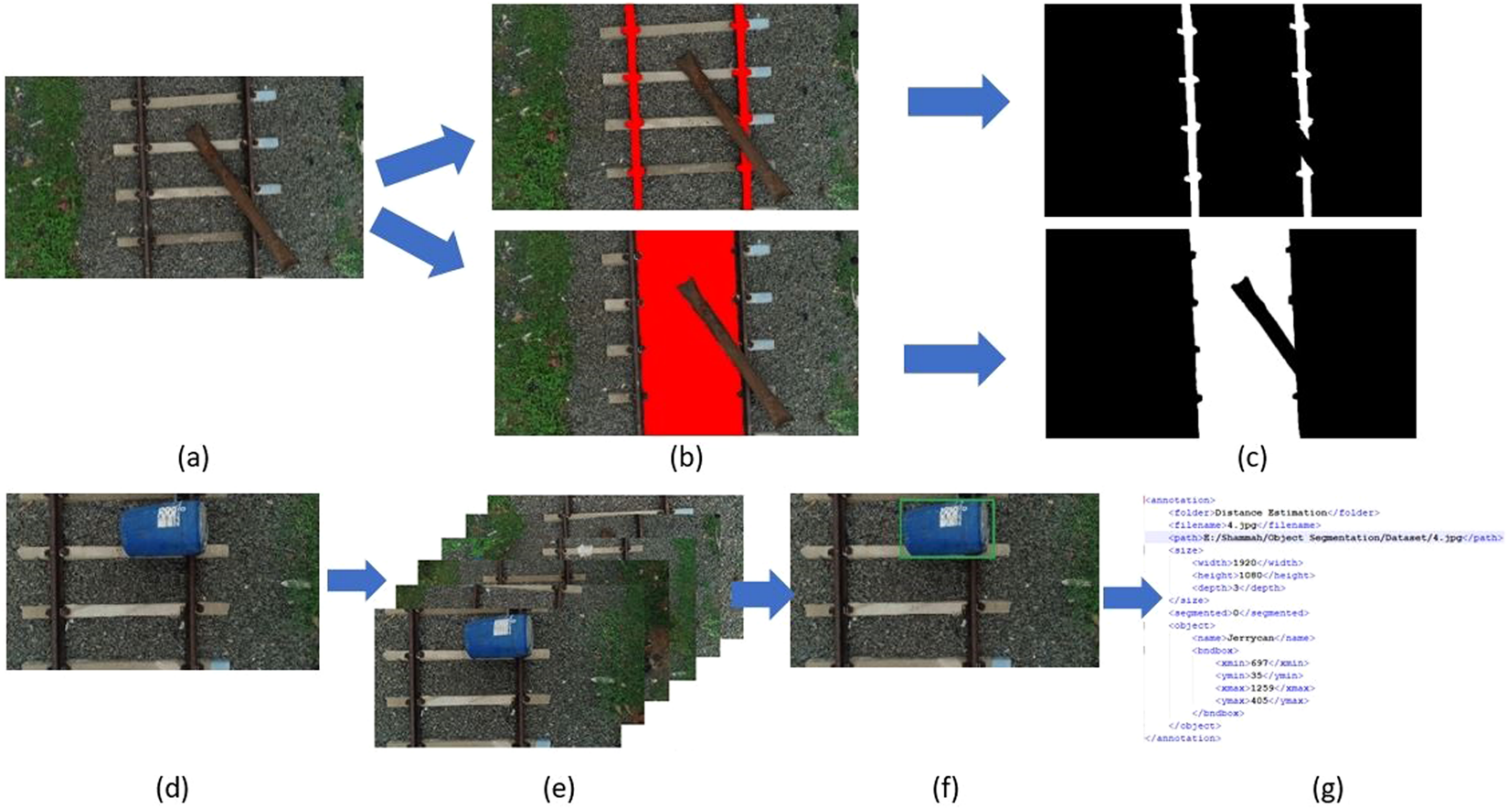
(**a**–**c**) Data Annotation Steps considered for Railroad Segmentation, where (**a**) Raw UAV Railroad Image (**b**) Labelling the Image as Rail and Gauge (**c**) Masking the Labelled Image for generating ground truth Images (**d**–**g**) Data Annotation Steps considered for Railroad Obstacle Detection, where (d) Original raw image (**e**) Augmented images (**f)** Bounding Box labelling (**g**) Obtained PASCAL VOC file.

The annotation of the dataset is intended for railroad semantic segmentation and obstacle detection. Figure 3 illustrates the annotation procedures for railroad segmentation and obstacle detection, respectively. Authors from the Computer Science and Engineering department developed comprehensive annotation guidelines to guarantee accuracy and consistency in the annotations. Six undergraduate project students from the group serve as the primary annotators. They provide feedback to the authors and may encounter a variety of scenarios while annotating. After familiarizing themselves with the annotation rules, each annotator begins the process of initial data annotation. Once the annotations are completed, the data is thoroughly examined by the authors of this work. Authors reannotate problematic images if they find the annotations inadequate. This iterative process guarantees the superior quality and consistency of the annotated data. Upon the completion of the annotation procedure, the finished picture data and associated annotation files are obtained. During the data annotation phase, two distinct and separate image annotation processes were carried out to enhance the dataset’s utility for different applications. The first annotation process focused specifically on railroad semantic segmentation, while the second was dedicated to obstacle detection.

**Fig. 4:**
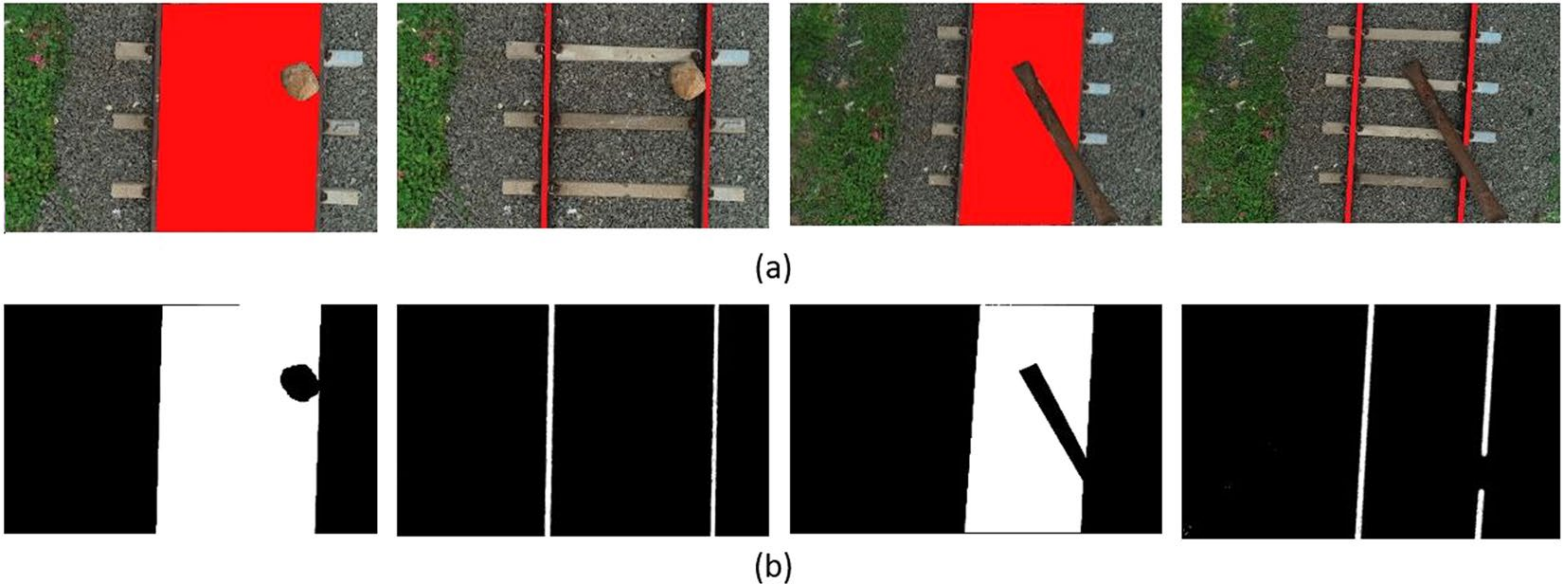
Sample data annotations for railroad semantic segmentation where, (**a**) Labelling (**b**) Masking.

In the first category of image annotation, each image is meticulously labeled to produce a binary mask that delineates both the rail and gauge. This process involves using Adobe Photoshop, where the images are assigned to two specific classes: rails and gauge. These classes are integral to the segmentation task, allowing for the extraction of railroad elements and determining the proximity of obstacles to the rail tracks. A binary mask is generated from the labelled images using Matlab’s colour threshold module. Figure 4 displays an illustration of labeled and masked image samples of the railroad segmentation process.

**Fig. 5:**
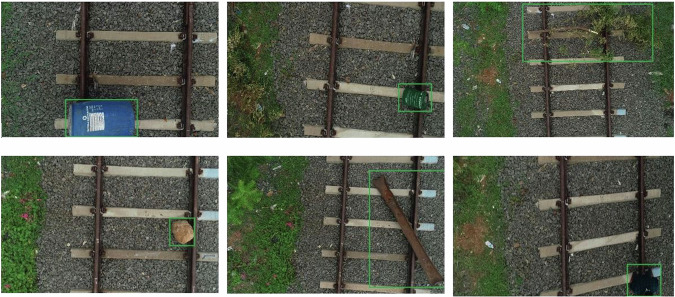
Sample data annotations for railroad obstacle detection.

In the second category, there are two phases involved in preprocessing data for obstacle detection: data augmentation and data annotation. Initially, the dataset available for this task is often relatively small, which can be inadequate for the effective training and testing of deep neural networks. Through a variety of geometric transformation techniques, the initial collection of 315 images was increased to a total of 2002 images, six times its original size. The data annotation results, shown in Fig. 5, can be employed for the implementation of railway obstacle identification. Labelme is a software application utilised to annotate railway images in order to locate obstacles.

### Data augmentation

**Fig. 6:**
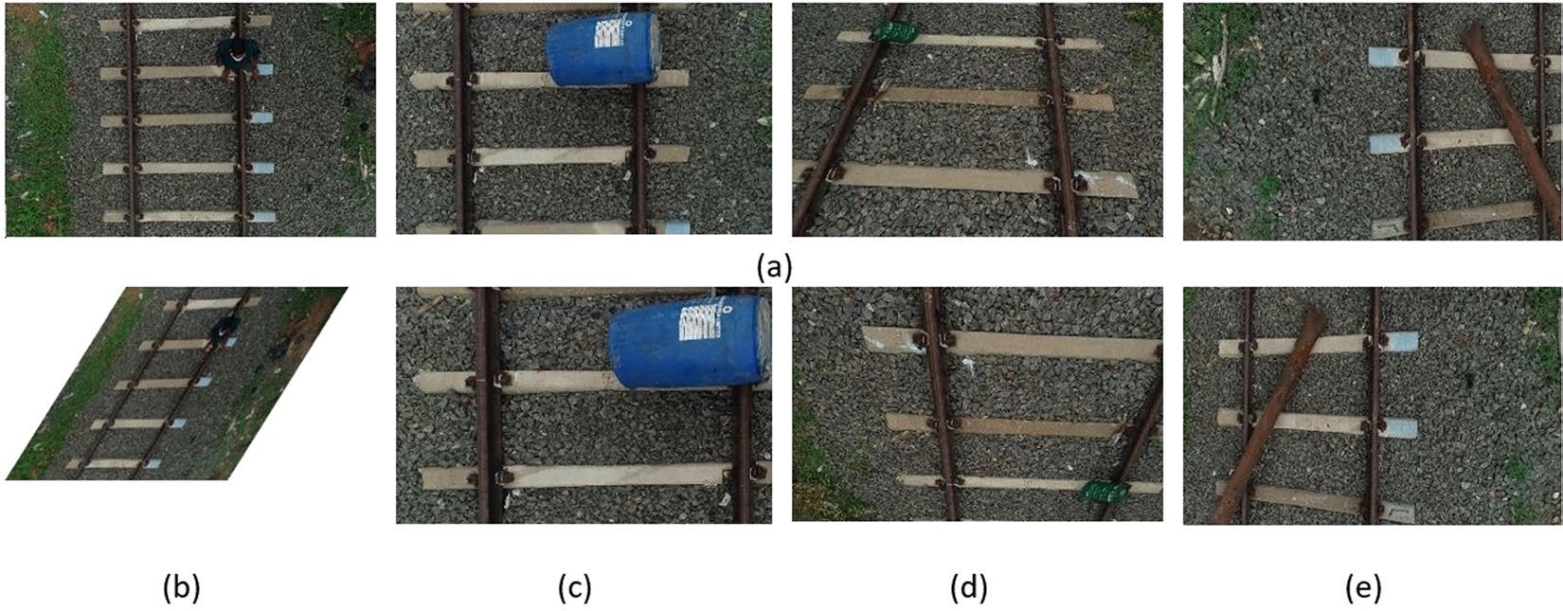
Outcomes of Data Augmentation (**a**) Raw Images (**b**) Image after Shear (**c**) Image after Zoom In (**d**) Image after Rotate (**e**) Image after Horizontal Flip.

Data augmentation is implemented using the ImageDataGenerator class from the Keras package^21^. Figure 6 depicts several augmentation processes considered for various classes, including zoom (0.2), brightness (0.5, 1.5), shear (0.2), rotation (40, 60), and horizontal flip. The annotation of data using the LabelImg tool for labeling and bounding box construction follows the augmentation procedure. According to Zhao *et al*.^22^, a bounding box must be produced to determine the precise location of the desired obstacles, containing six distinct labelings such as jerry can, human, iron rod, barrel, branch, and boulder in this dataset. To ensure the dataset is suitable for training and testing detectors, two different bounding box formats have been created: pascal_voc, (which supports all deep neural network models except YOLO), so separate labeling is done for YOLO models and updated in the dataset. To train the latest object detection models, TensorFlow records and label map documents are also generated.

**Fig. 7:**
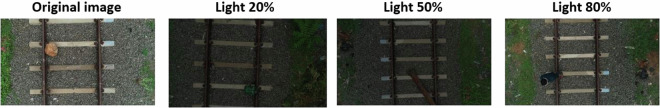
Annotations of different light conditions for railroad obstacle detection.

In challenging environmental conditions, a reliable deep neural network model for obstacle detection can be developed for railroad applications. One of the main issues in this railroad application is the influence of lighting. To evaluate the effectiveness of obstacle detection under various lighting conditions, four distinct light illuminations are considered using the brightness parameter in the augmentation process. The original image, light at 20%, light at 50%, and light at 80% are the light effects. The annotation outcomes for the different lighting situations are shown in Fig. 7.

## Data Records

**Fig. 8:**
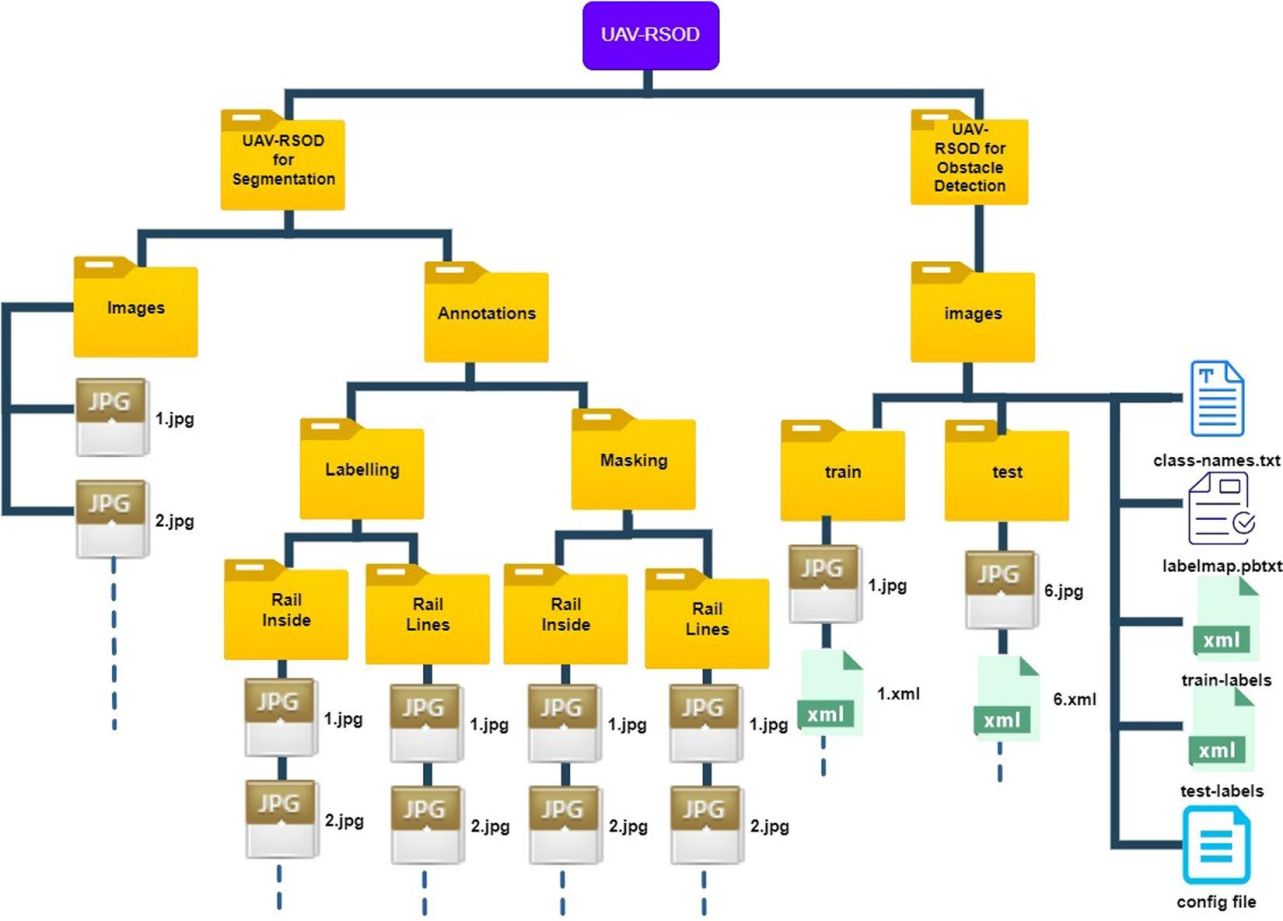
Organization of the UAV-RSOD Dataset File Directory.

The UAV-RSOD dataset is available on Zenodo^23^ in a zip file in accordance with the data specifications. In total, the file occupies 1.55 GB of disk space. The UAV-RSOD dataset is organized into two main directories, as depicted in Fig. 8. The first directory, V1_UAV-RSOD, is dedicated to segmentation tasks, while the second directory, V2_UAV-RSOD, focuses on obstacle detection. The V1_UAV-RSOD for Segmentation folder contains two subfolders: Images and Annotations. The images directory organises unprocessed images taken by UAVs, and the Annotations folder is further categorized into two subfolders, namely Labeling and Masking. The dataset includes both binary masks for deep learning applications and labeled visualizations that overlay masks on the original images. The labeled visualizations facilitate data exploration and quality control, providing immediate insights into annotation quality. Users focused on model training may opt to utilize only the binary masks, with the option to download this subset separately, minimizing storage requirements.

The Labeling folder has two subfolders called Rail Inside and Rail Lines, where both contain all the rail inside labeled images and all the rail border labeled images, respectively. These two folders are considered under Masking, the parent folder of Rail Inside and Rail Lines folders, where all the rail inside binary mask images and all the rail border binary mask images are available. In total, each subfolder of Labeling and Masking contains 630 labeled and 630 masked images.

In the V2_UAV-RSOD for Obstacle Detection folder, there are two subfolders named ‘train’ and ‘test’. According to Google’s best practices, 20% of the 400 images with a resolution of 1920 × 1080 pixels are allocated for testing, while 80% of the 1602 images with the same resolution are designated for training^24^. In addition to these, five more files are provided for obstacle detection: a class-names text file, a label map file, train labels, test labels, and a deep neural network model configuration file.

**Fig. 9:**
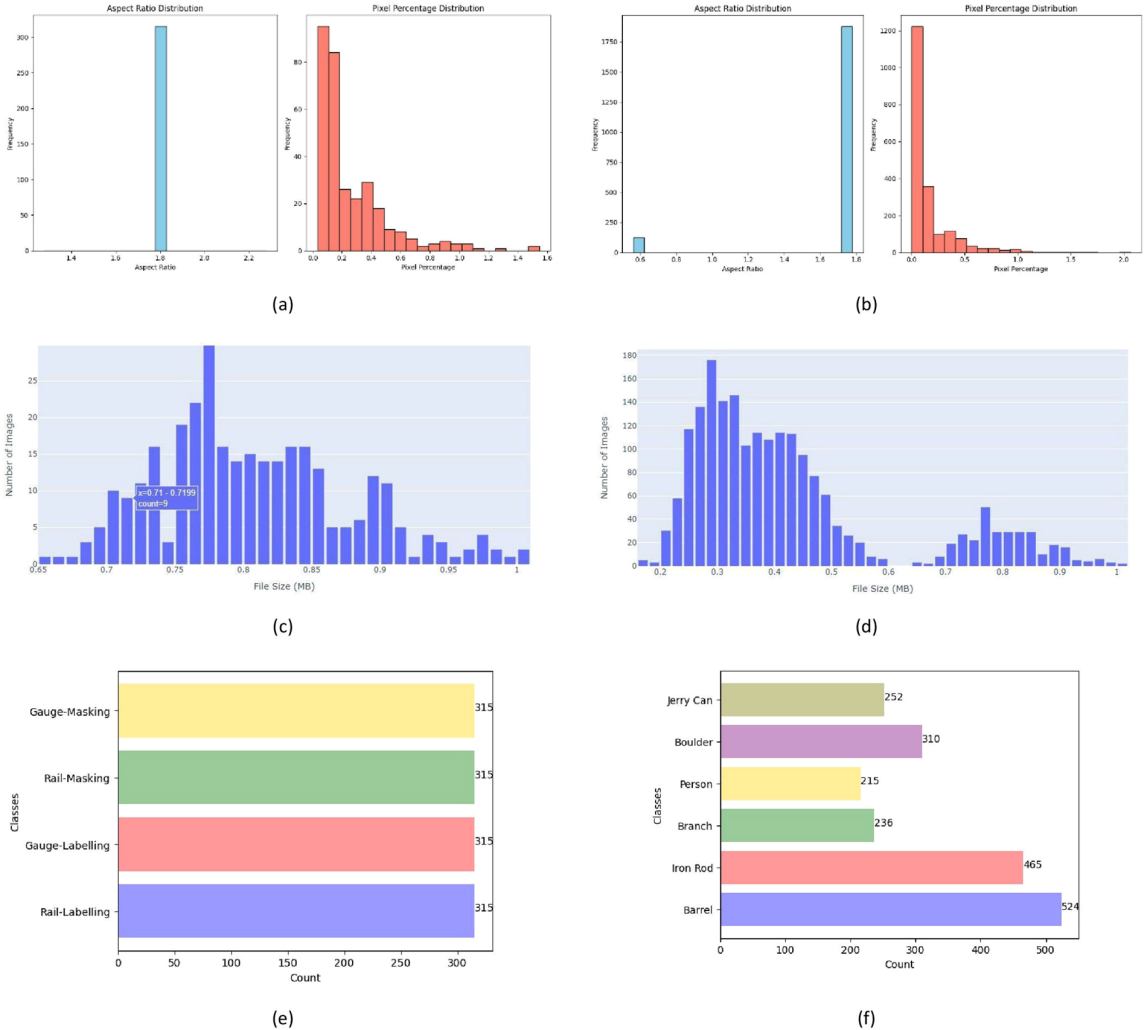
Comprehensive view of UAV-RSOD dataset characteristics where (**a**) shows aspect ratio & pixel percentage histogram of images for semantic segmentation, (**b**) illustrates aspect ratio & pixel percentage histogram of images for obstacle detection, (**c**) displays distribution of image file size for semantic segmentation, (**d**) shows distribution of image file size for obstacle detection, (**e**) labelled classes distribution for semantic segmentation and (**f**) labelled classes distribution for obstacle detection.

Table 2 offers statistical information of value for the UAV-RSOD dataset, while Fig. 9 illustrates the key statistics of the UAV-RSOD dataset, such as aspect ratios, pixel percentages, distribution of image file sizes, and number of annotations per class for railroad semantic segmentation and obstacle detection, respectively. The aspect ratio describes the proportional relationship between the width and height of an image and Pixel percentage refers to the proportion of pixels that a particular element or area occupies within a given space. In the left graph of Fig. 9(a) shows aspect ratio distribution which is a single prominent blue bar at the aspect ratio value of 1.6 with a frequency just above 300 and the right graph of Fig. 9(a) displays pixel percentage distribution with multiple red bars decreasing in height from left to right, starting at a pixel percentage of 0 and ending at around 1.6, with the highest frequency being just under 80 at the pixel percentage value closest to 0. In the left graph of Fig. 9(b) shows aspect ratio of 1.0 which is the most common, occurring more than 1750 times, while other aspect ratios (0.6 and 1.6) are rare and the right graph of Fig. 9(b) displays pixel percentage distribution in which Lower pixel percentages (0 and 0.5) are more common, with the highest frequency observed at a pixel percentage of 1. Higher pixel percentages (around 1.5 to just under 2) are rare.

**Table 2:** UAV-RSOD Dataset Key Statistics.

Types of UAV-RSOD	Metric	Mean	Standard Deviation	Minimum	Maximum	Median
Semantic Segmentation	Pixel Percentage	1.0	0.0	1.0	1.0	1.0
	Aspect Ratio	1.77	4.44e-16	1.77	1.77	1.77
Obstacle Detection	Pixel Percentage	1.0	0.0	1.0	1.0	1.0
	Aspect Ratio	1.701	0.2940	0.5625	1.77	1.77

## Technical Validation

The objective of semantic segmentation, a fundamental problem in computer vision, is to assign a label to each pixel in to a picture. Rail, gauge, and background are the three classes considered for semantic segmentation in the UAV-RSOD dataset, which is used to accomplish railroad extraction using semantic segmentation. Likewise, one of the primary goals of the computer vision method for identifying object instances in images is obstacle detection. The UAV-RSOD dataset, used to conduct railroad obstacle detection, includes six classes: iron rod, jerry can, barrel, human, boulder, and branch.

### Training details of semantic segmentation

We evaluated the effectiveness of four state-of-the-art semantic segmentation models, namely PSPNet^25^, SegNet^26^, RSNet^27^, and Lite-RSNet^2^, using the UAV-RSOD dataset. The models’ performance is greatly influenced by the learning rate, which varies from 1 × 10^−3 to 1 × 10^−4. The training hyperparameters include the 1,000 epochs needed to train the dataset. All models were trained using training and test datasets with sizes of 80% and 20% correspondingly. Every segmentation model was developed using the Keras library, in conjunction with TensorFlow as the underlying technology. A Windows PC equipped with an NVIDIA GeForce RTX 2070 GPU and an Intel i7 CPU was used to ease the training of the models.

### Semantic segmentation evaluation

**Fig. 10:**
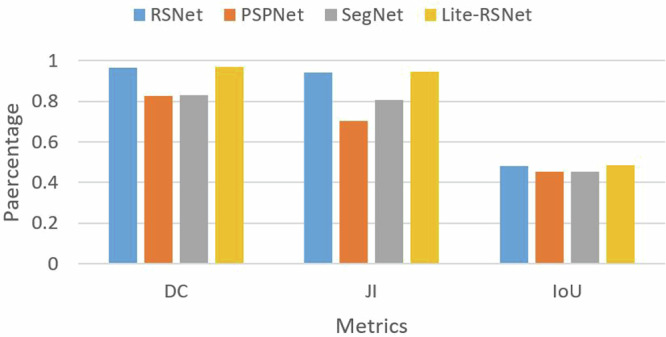
Comparison of various models in terms of semantic segmentation performance metrics.

Figure 10 presents a comparison of the validation outcomes for the performance evaluation metrics. Evaluation of the proposed segmentation models is conducted using semantic segmentation performance indicators such as the Dice Coefficient (DC), Intersection over Union (IoU), and Jaccard Index (JI). Due to the pixel-wise segmentation nature of railway semantic segmentation, which is more similar to a classification problem, these evaluation metrics are suitable for this specific kind of semantic segmentation. The terms ‘true positives’, ‘false negatives’, and ‘false positives’, which represent correctly detected railroad pixels, ignored railroad pixels, and mistaken pixels, respectively, are used to describe the evaluation criteria. All the models provide adequate measures in the segmentation evaluation for the UAV-RSOD dataset; especially, the Lite-RSNet model achieves a Dice Coefficient of 0.972 and a Jaccard Index of 0.945, respectively, which demonstrates the robustness of the dataset.

**Fig. 11:**
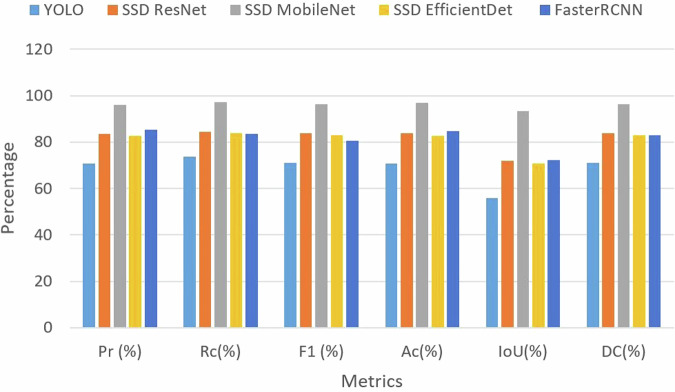
Visual results of the self-constructed UAV-RSOD test set for several models. From left to right are predicted results of input images, ground truth images, PSPNet, SegNet, RSNet, and Lite-RSNet models.

The chosen models all provided better results and suitable boundary segregation. The color palette displayed at the bottom of the image serves as a significant reminder of the successful division of the expected images into different colored categories for the background, rail, and gauge. This is illustrated in Fig. 11.

### Training details of obstacle detection

The deep neural network models utilized in this research require extensive training. Every setting is carried out using an Anaconda prompt, and Colab Pro was used to complete the model training for railroad obstacle identification in our study. Additionally, Jupyter Notebook is utilized to evaluate the photos using the obtained inference graph. Colab Pro, equipped with a Tesla 4 GPU and 25 GB of RAM, was used for the training. Yolov5 is the only model not selected from the most recent TensorFlow 2 detection model zoo^28^. The learning rates chosen for the various deep neural network models implemented in this evaluation were 1e-3 for Yolov5 and 8e-3 for the remaining models. All models were identically assigned a momentum value of 0.9. The batch size is stipulated as 2 based on the amount of sample data transmitted over the network model. The optimizers were selected for each model based on variations in accuracy and loss across different epochs. Every model made use of a momentum optimizer. Weight decay is a crucial mechanism employed to mitigate the excessive growth of weights. This is achieved by multiplying the weights by 0.99 after each update.

### Obstacle detection evaluation

**Fig. 12:**
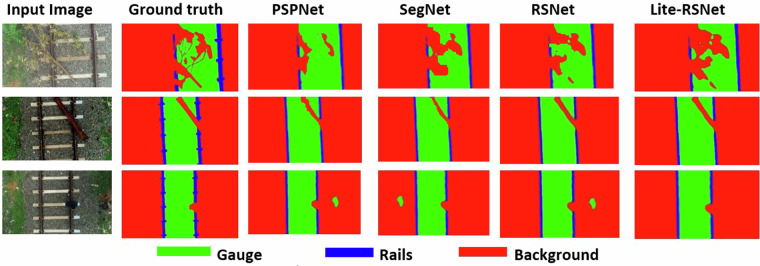
Comparison of various models in terms of obstacle detection performance metrics.

The quantitative evaluation metrics for training several models of railroad obstacle detection are shown in Fig. 12 and include Precision (Pr), Recall (Rc), F1-Score (F1), Accuracy (Acc), DC, and IoU. The SSD MobileNet model yielded a higher success ratio, as seen in Fig. 12. However, when compared to other models, YOLOv5 produced lower accuracy. The SSD MobileNet model achieved 95.89% precision, 97.22% recall, 96.41% F1-score, 96.75% accuracy, 93.18% intersection over union, and 96.41% Dice Coefficient. Specifically, the accuracies achieved were 70.83% (YOLOv3), 83.75% (SSD ResNet), 96.75% (SSD MobileNet), 77.25% (SSD EfficientDet), and 84.75% (Faster RCNN).

Railroad obstacle detection accuracy is measured using Mean Average Precision (mAP). Table 3 displays selected deep neural network models’ mAP results on the UAV-RSOD dataset. The accuracy recall (AR) of MobileNet, as measured by mean average precision (mAP) scores at IOU = 0.5, is around 2% greater than FasterRCNN and between 30% and 50% greater than YOLOv5. Under more challenging test scenarios with IOU values ranging from 0.50 to 0.95, and at IOU = 0.75, MobileNet outperforms Faster RCNN by 2% in terms of mAP and YOLOv5 by 20% to 50%. When compared to all other models, MobileNet yields a greater success percentage in terms of mAP and AR.

**Table 3:** Evaluation of different obstacle detection models via mAP.

Models	YOLO	SSD ResNet	SSD MobileNet	SSD EfficientDet	Faster RCNN
IoU and Loss					
mAP (%) 0.50:0.95 (all-100)	49.45%	44.75%	52.90%	46.00%	51.30%
mAP (%) 0.50 (all-100)	81.05%	73.70%	84.40%	77.70%	82.70%
mAP (%) 0.75 (all-100)	54.10%	47.80%	58.70%	49.50%	55.30%
mAP (%) 0.50:0.95 (small-100)	10.00%	10.00%	10.00%	10.00%	10.00%
mAP (%) 0.50:0.95 (medium-100)	39.05%	28.50%	35.30%	42.80%	33.20%
mAP (%) 0.50:0.95 (large-100)	49.70%	46.15%	53.50%	45.90%	52.40%
AR (%) 0.50:0.95 (all-1)	58.50%	56.95%	60.00%	57.00%	58.10%
AR (%) 0.50 (all-10)	67.05%	66.50%	69.80%	64.30%	67.50%
AR (%0.75) (all-100)	68.95%	67.80%	71.90%	66.00%	69.10%
AR (%)0.50:0.95 (small-100)	10.00%	10.00%	10.00%	10.00%	10.00%
AR (%)0.50:0.95 (medium-100)	58.10%	36.35%	52.50%	63.70%	47.30%
AR (%)0.50:0.95 (large-100)	68.25%	68.85%	70.40%	66.10%	69.60%
Localisation Loss	0.2002675	0.98795	0.181667	0.218868	0.188975
Classification Loss	0.584797	0.587304	0.7041	0.465494	0.277024
Regularisation Loss	0.080503	0.20223	0.116207	0.044799	0.288253
Total Loss	0.865567	0.686099	1.001973	0.729161	0.754252

**Fig. 13:**
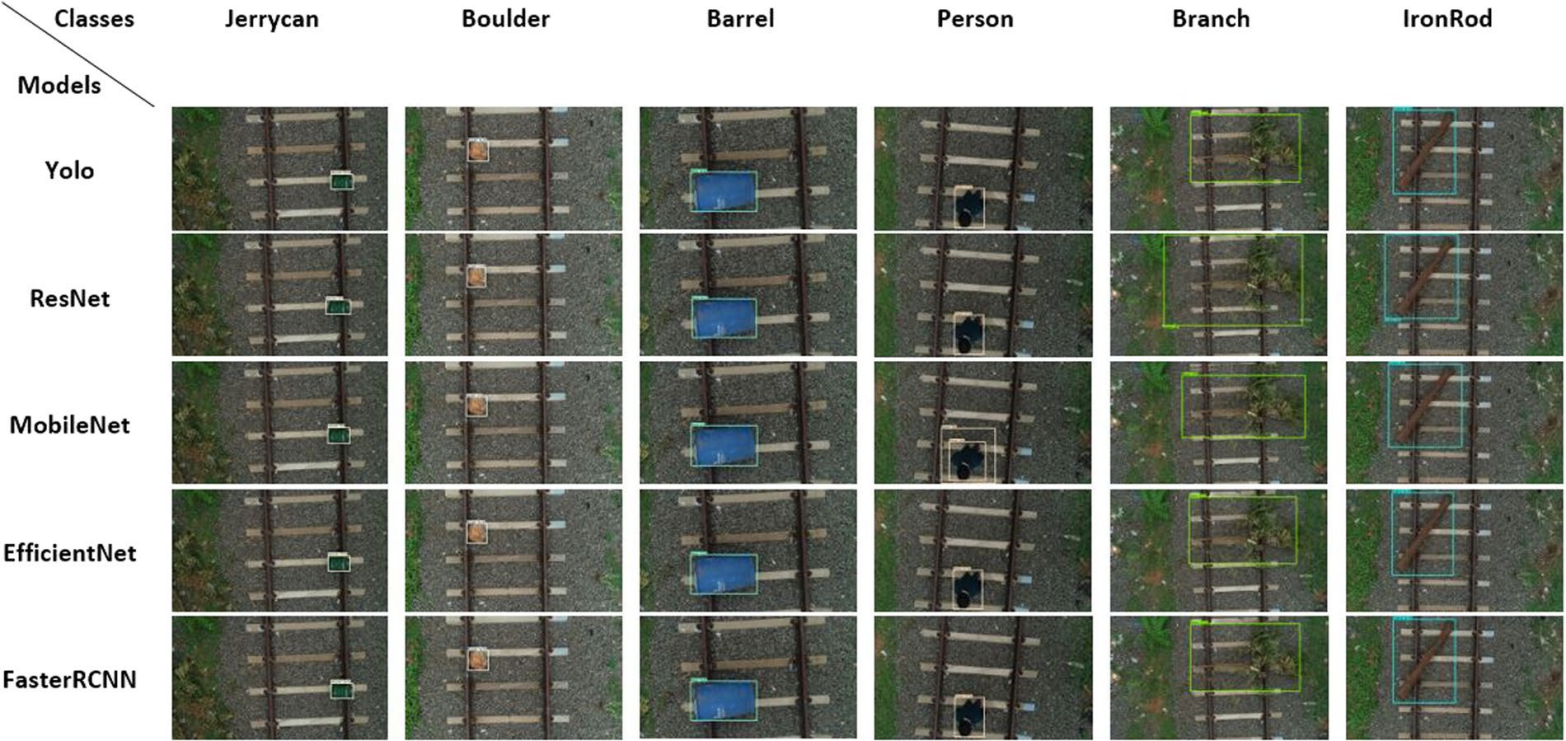
Visual outcomes of various obstacle detection models for UAV-RSOD Dataset.

Figure 13 displays a graphical exposition of the railway obstacle detection for all six classes, utilising the deep neural network methods examined in this work. The UAV-RSOD is the source of all test aerial railroad images. Thus, adequate obstacle detection outcomes lead to the acceptance of the UAV-RSOD dataset as a benchmark level.

### Analysis of data collection

Traditional methods of monitoring and detecting obstructions on railways, which rely on human observation or rail inspection vehicles, generally collect images from a considerable distance from the ground^29^. This limitation leads to a scarcity of image data, which poses challenges in training models appropriately. When compared, the application of UAVs for railroad monitoring and obstacle detection offers significant advantages over traditional methods, which are more time-consuming and costly^30^. Additionally, the adoption of UAVs enhances environmental sustainability by minimizing the ecological footprint of railroad monitoring activities. This innovative approach not only streamlines the process but also has the potential to substantially lower overall railroad maintenance costs.

In order to train deep learning models, railroad images were captured by a UAV equipped with a high-resolution camera in this study. Appropriate flying parameters were utilized to ensure the best image quality. The UAV’s photographs are captured at a lower altitude and cover obstructions, hence reducing the number of train accidents. Conversely, alternative railroad photographs are captured from a closer viewpoint using a ground camera^31,32^. The study also includes images of railroad obstacle detection in a range of lighting scenarios, which enhances the model’s capacity to generalize. In addition, the dataset provides annotations for training obstacle detection using both YOLO and PASCAL VOC formats. This dual-format strategy simplifies data processing for researchers and allows developers to concentrate more on enhancing their algorithms. The dataset categorizes railroad obstacle detection into six distinct obstacle classes. Researchers can employ this dataset to investigate advanced algorithms for UAV image detection and to design sophisticated train inspection systems that employ UAV technology.

### Limitations

Note the following problems encountered when operating the drone, which made data collection difficult:

- **Security issue:** The Indian Railways have numerous regulations on the use of drones for taking pictures, which raises security concerns. Restrictions on the usage of drones persist even with a letter of authorization from the security authorities.
- **Ecological concerns:** The drone’s flight altitude was limited by potential hazards such as electrical wires, security sites, and other establishments emitting lasers.
- **Manual threats:** There have been several incidents where adults and children threw stones at the drone, particularly when it was operating far from its base.
- **Insufficient variety of obstacles:** The majority of barriers on railroads consist of rocks, tree branches, people, and landslides, with relatively few animal interruptions. Ensuring class balance was therefore difficult.
- **Concerns pertaining to ethics, privacy, and the law:** Issues include illicit data acquisition, invasions of privacy, and unauthorized photography.
- **Cybersecurity-related problems:** These include information theft on critical infrastructure and illegal access to private communication networks.
- **Physical issues:** Unmanned Aerial Systems (UAS) flying through restricted airspace and flight pathways can cause collisions with drones and piloted aircraft, potentially damaging vital assets or injuring persons.
- **Breaching safe boundaries:** This includes crossing safe boundaries, such as those of foreign states or military installations.
- **Geographical Bias and Generalizability:** The UAV-RSOD dataset primarily focuses on objects commonly found in South India, such as Jerrycan, Boulder, and Barrel, which introduces a regional bias. As a result, models trained on this dataset may perform better in detecting these specific objects within similar environments but may face challenges when applied to different regions with varying object types and characteristics. This geographical bias could limit the generalizability of the models to other contexts.

## Data Availability

The augmentation code is referred and clarified in Data Augmentation section. However, we released and shared the code for model evaluation (https://gist.github.com/dsabarinathan/782fdb7b10bc4ac1424845662892120e).
